# H-Wave^®^ Device Stimulation: A Critical Review

**DOI:** 10.3390/jpm11111134

**Published:** 2021-11-02

**Authors:** Tyler K. Williamson, Hugo C. Rodriguez, Andrew Gonzaba, Neil Poddar, Stephen M. Norwood, Ashim Gupta

**Affiliations:** 1 University of the Incarnate Word School of Osteopathic Medicine, San Antonio, TX 78209, USA; tylerwill33tamu@gmail.com; 2 Department of Orthopaedic Surgery, NYU Langone Medical Center, New York, NY 10016, USA; 3 Department of Orthopaedic Surgery, Holy Cross Orthopedic Institute, Fort Lauderdale, FL 33334, USA; hcrodrig2112@gmail.com; 4 Ross University School of Medicine, Miramar, FL 33027, USA; andrewgonzaba@mail.rossmed.edu; 5 Future Biologics, Lawrenceville, GA 30043, USA; neilpoddar11@gmail.com; 6 Retired Orthopaedic Surgeon, Austin, TX 78738, USA; norwoods@austin.rr.com

**Keywords:** pain, analgesia, neurogenic, electrotherapy, neuromuscular stimulation, H-Wave

## Abstract

Pain treatments have historically centered on drugs, but an “opioid crisis” has necessitated new standards of care, with a paradigm shift towards multi-modal pain management emphasizing early movement, non-narcotics, and various adjunctive therapies. Electrotherapies remain understudied and most lack high-quality clinical trials, despite a desperate need for effective adjunctive options. A systematic search of human clinical studies on H-Wave^®^ device stimulation (HWDS) was conducted as well as a comprehensive review of articles articulating possible HWDS mechanisms of action. Studies unrelated to H-Wave were excluded. Data synthesis summarizes outcomes and study designs, categorized as pre-clinical or clinical. Pre-clinical studies demonstrated that HWDS utilizes a biphasic waveform to induce non-fatiguing muscle contractions which positively affect nerve function, blood and lymph flow. Multiple clinical studies have reported significant benefits for diabetic and non-specific neuropathic pain, where function also improved, and pain medication usage substantially dropped. In conclusion, low- to moderate-quality HWDS studies have reported reduced pain, restored functionality, and lower medication use in a variety of disorders, although higher-quality research is needed to verify condition-specific applicability. HWDS has enough reasonable evidence to be considered as an adjunctive component of non-opioid multi-modal pain management, given its excellent safety profile and relative low cost. Level of Evidence: III.

## 1. Introduction

Chronic pain is one of the most common primary care complaints, affecting 13–15% of adults in the United States [1]. Federal and state health care costs for management of pain exceeds $100 billion annually, much more than the combined cost of treatments for cancer, diabetes, and heart disease [2,3]. Pain has generally been categorized based on stimulus origin, ranging from common musculoskeletal nociception to diabetic and non-specific neuropathies, although it is often a symptom of an underlying pathologic condition. Musculoskeletal and neuropathic pain is all too commonly treated as the direct regimen target, with less emphasis towards the pain generating source [4]. Opioids, non-steroidal anti-inflammatory drugs (NSAIDS), and acetaminophen, as well as physical and occupational therapy, have all been heavily utilized. Opioid medications hyperpolarize neurons, leading to a decrease in neuronal excitability and diminished pain sensation [5]; however, adverse effects including sedation, respiratory depression, constipation, nausea, and dependence are all problematic [6]. NSAIDs also have proven efficacy in chronic pain reduction by reducing synthesis of prostaglandins and other mediators, providing both analgesic and anti-inflammatory benefits, albeit with the downside of prostaglandin reduction in other organ systems, most notably contributing to gastric ulcer formation and decreased renal perfusion [5]. Acetaminophen is another analgesic and antipyretic that inhibits various COX pathways within the central nervous system [7], also having potentially adverse effects, primarily related to over dosage, including hepatoxicity, hypersensitivity, nephrotoxicity, electrolytes abnormalities, and even death [8,9].

Various modalities, often applied in physical and occupational therapy regimens, have some demonstrated effectiveness as adjunctive, less harmful treatments for pain. While direct benefits can be achieved from physical activity and exercise alone, a significant proportion of adults worldwide remain inactive, with 31% failing to meet recommended daily physical activity levels [10]; furthermore, 65% of pain patients become non-adherent to prescribed home exercise regimens [11]. It has become increasingly critical to incorporate several different modalities into practical pain treatment plans to address aspects not fully covered by one single therapy [12,13]. More efficacious modalities with limited side-effect profiles are desperately needed that target multiple levels in pain generation and transmission. Proposed electrotherapies, applied as adjuncts or as stand-alone treatments, have included Transcutaneous Electrical Nerve Stimulation (TENS), Microcurrent Electrical Nerve Stimulation (MENS), Interferential Current Stimulation (ICS), Neuromuscular Electrical Stimulation (NMES), Percutaneous Electrical Nerve Stimulation (PENS), Percutaneous Neuromodulation Therapy (PNT), and H-Wave^®^ device stimulation (HWDS).

H-Wave device stimulation (HWDS) is a form of transcutaneous electrotherapy that utilizes a specific proprietary waveform to stimulate contractions in muscle fibers, resulting in increased blood flow and decreased edema. Other mechanisms of action include nitric oxide dependent vasodilation and angiogenesis with repeated use [14,15]. These physiologic responses occur through application of a biphasic, exponentially decaying waveform using a low frequency and long pulse duration, causing a non-fatiguing, low-tension contraction which mimics natural voluntary contractions [16,17]. This contrasts with the mechanism of other electrotherapies, which often create fatiguing, tetanizing contractions, usually achieving only short-term relief. The minimal exertion requirements of HWDS are without reported side effects, presenting a viable treatment option for patients with various comorbidities or compromised mobility status [16,17]. At a 1000 ohm load HWDS delivers a current between 0 and 35 mA and a voltage between 0 and 35 V, a pulse duration up to 5 ms and treatment components of 2 and 60 Hz [16,17]. HWDS is a relatively low-cost, mobile treatment modality, requiring limited instruction for effective use. Flexible placement of self-adhesive electrodes permits applicability to most body parts, allowing patient-specific optimization of treatment parameters [18]. HWDS has the potential to be a mainstay treatment option for neuropathic and musculoskeletal pain, with a fair amount of peer-reviewed clinical evidence already reported for an assortment of pain conditions.

The primary objective of this systematic review of available HWDS clinical studies is to appraise the degree of pain relief, improvement in functionality, and reduction in analgesic medication use for various pain conditions. Additionally, pre-clinical HWDS data regarding physiological effects and proposed cellular mechanisms of action will be comprehensively reviewed. Finally, study weaknesses, inconsistencies, and remaining uncertainties regarding clinical effectiveness will be identified and discussed in order to recommend strategies to improve methodology and reporting for future HWDS studies.

## 2. Materials and Methods

### 2.1. Design of This Study

This critical review included case series, primary trials, reviews and meta-analyses, reporting on H-Wave therapy in treating pain and decreased function, along with non-clinical studies emphasizing HWDS physiologic processes. All available studies meeting eligibility criteria were included, regardless of publication date.

#### Eligibility Criteria

Studies meeting eligibility criteria included those applying to utilization of H-Wave therapy in treatment of acute, chronic, and post-surgical musculoskeletal pain and function loss, as well as any articles articulating possible HWDS mechanisms or effects. Literature which did not specifically relate to HWDS was excluded from eligibility. Human studies of adult participants (ages ≥ 18 years) were included in the systematic review, regardless of geographic origin, while animal model studies were included to review pre-clinical basic science data. Accepted clinical studies considered H-Wave therapy used as an intervention with the aim of relieving pain, improving functionality, and/or decreasing pain medication use, whether used alone or as part of a pain treatment regimen. Since there are two settings implemented with HWDS, using a unique biphasic exponentially decaying wave, both frequencies were utilized and reported, ultra-low (2 Hz) and high (60 Hz). This review included studies comparing standard-of-care treatments for musculoskeletal pain and decreased functionality with or without use of H-Wave therapy. The primary outcomes are a global evaluation of H-Wave therapy and whether it can subjectively decrease pain, reduce medication use and improve functionality in patients with musculoskeletal pain and dysfunction.

### 2.2. Information Sources

A systematic search was conducted in PubMed, ScienceDirect, PEDro, Web of Science, Scopus, EMBASE and Google Scholar databases of English language HWDS articles published before June 2021. General reviews and related articles were used as a secondary search to include any eligible studies reported from reference lists not already included.

### 2.3. Search Strategy

The PRISMA 2020 checklist and flow diagram were used as the eligibility and inclusion criteria during the search and selection process. A web-based reference software system (RefWorks) was used for data management [19,20].

### 2.4. Selection Process

Titles and abstracts were initially reviewed by two separate reviewers when selecting studies. These findings were uploaded to the web-based reference software system. A second review was conducted by a different reviewer to verify that the studies met the eligibility criteria. A third reviewer served the role of discussing any discrepancies found by the first two reviewers.

### 2.5. Data Collection Process

After articles were fully assessed to meet inclusion criteria, two separate reviewers extricated the available data, including study characteristics, specific application of H-Wave therapy, outcome measurements, and clinical implications.

### 2.6. Data Items

Relevant data points and study characteristics including authors, study design, and publication year were extracted, along with unique characteristics of H-Wave therapy, frequencies used, electrode placement locations, voltage applied, duration of device use, and specific musculoskeletal pain and dysfunction treatment. Outcome measures related to a reduction in pain and medication use, as well increased functionality, were reported in percentages and absolute values where available. Measures included for a reduction in pain and improved functionality included subjective patient pain scale, specific functions, activities of daily living (ADLs), and passive and active range of motion testing, with variables reported from any time interval. For pre-clinical research, relevant physiological findings and measures were included.

### 2.7. Risk of Bias and Methodological Weaknesses

The risk of study biases and methodological weaknesses were identified and stratified under “Clinical Evidence Certainty” as low, moderate, and high. Ten domains included selection bias, performance bias, detection bias, attrition bias, and reporting bias; seven domains from the Risk Of Bias In Non-randomized Studies of Interventions (ROBINS-I) tool were used for quasi-randomized and observational studies [21]; the Risk of Bias 2 (RoB 2) tool evaluated randomized control trials [22]. After an independent assessment of each study from two reviewers, consensus risk levels of bias and study weaknesses were derived through interactive discussion.

### 2.8. Data Synthesis

Data synthesis was performed, followed by subgroup analysis if needed, due to a variety of studies included pertaining to the H-Wave therapy used, diagnoses being treated, frequency used (ultra-low or high), and treatment population and indications. Outcome measures and study designs have been summarized under results, categorized as pre-clinical or clinical data.

## 3. Results

### 3.1. Search Results

**Figure 1:**
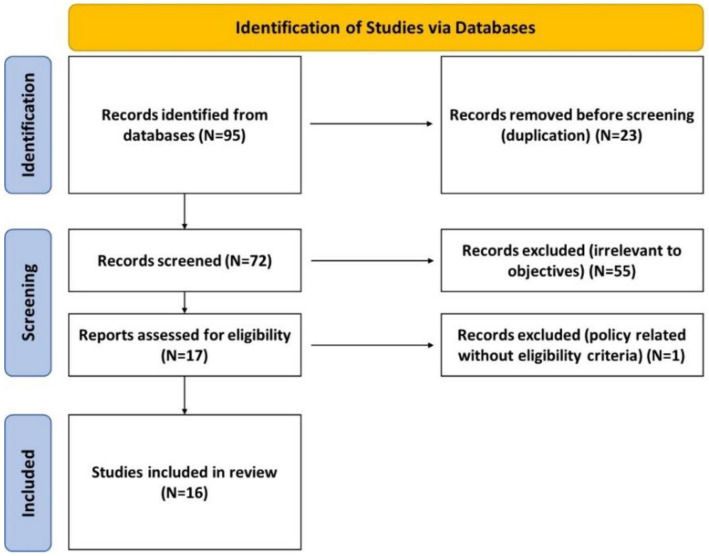
PRISMA flowchart.

The systematic searches retrieved 95 records; after screening titles and abstracts, 72 full-text assessments were completed; 16 studies were selected which were pre-clinical, clinical, or both. Of these, 9 clinical studies reported findings from 6789 patients. The PRISMA flowchart in Figure 1 illustrates the selection process [20], while all remaining HWDS studies have been summarized in Table 1.

### 3.2. Pre-Clinical Studies

Seven studies addressed the mechanism of action of HWDS from a basic science approach in the following categories: blood flow, muscle contraction, nerve action potential, and lymphatic function.

#### 3.2.1. H-Wave Technology

The H-Wave induces physiologic responses through utilization of a biphasic waveform. Kumar et al. stated the waveform is exponentially decaying [31]. The key characteristic which allows H-Wave to distinguish pain-origin targets involves its dual-frequency feature. The unit has two channels which propagate stimuli via four electrodes applied cutaneously targeting the pain site, as discussed by Julka et al. [18]. A hypothesis later tested by Blum et al. theorized that the H-Wave device can be employed at either ultra-low (2 Hz) or high (60 Hz) frequency to exert separate effects on muscle fibers or nerves, respectively [14,24].

#### 3.2.2. Blood Flow Effects

Four studies documented the effect of HWDS on peripheral blood flow. Smith et al. (2008) demonstrated vasodilatory effects from stimulation of skeletal muscle fibers by ultra-low-frequency H-Wave [14]. A companion study further applied a block with nitric oxide (NO), a potent vasodilatory compound, where the H-Wave stimulation subsequently had no added effect on microvascular diameter, strongly suggesting that H-Wave’s vasodilatory effects are, in part, NO mediated. Nitric oxide vasodilation may also play a separate role in increasing blood flow to pain-origin sites. Smith et al. (2011) conducted longitudinal H-Wave studies in hind limb rat musculature, eliciting 247% greater increase in blood flow relative to controls, which had initially been thought to be predominantly mediated by vasodilation [15]. Histological examination across 6 high-powered fields with BrdU stain revealed an average of 23 microvessels in the H-Wave-conditioned rat biceps femoris versus only 2 with the sham treatment. Increasing tissue blood flow and neovascularization may have important implications across various disease states. A related small case series by Blum et al. (2010) observed effects of H-Wave stimulation in three diabetic patients with chronic ankle venous stasis ulcers, reporting complete wound resolution by one, three, and nine months, respectively [25].

#### 3.2.3. Muscle Contraction Effects

The biphasic HWDS waveform allows penetration to muscle, causing a non-fatiguing, low-tension contraction. Blum et al. indicated that the contractions mimic voluntary, natural muscle contractions, with minimal exertion requirement [17]. The contraction generated by rhythmical, ultra-low-frequency HWDS simulates voluntary contraction of smaller, slow twitch skeletal muscle red fibers, as well as smooth muscle fibers of lymphatic vessels [14,24].

#### 3.2.4. Nerve Action Potential Effects

Blum et al. asserted that H-Wave therapy employed at high frequencies provides an accumulative, inhibitory effect on nerve action potentials through deactivation of sodium channel pumps [24], leading to a long-lasting analgesic effect from an accumulative post-synaptic depression. Blum et al. further suggested that HWDS does not trigger large white muscle fibers, delta sensory nerve fibers, or C pain nerve fibers, ultimately negating the painful effects of tetanizing fatigue. Tsang et al. (1998) had previously performed a rat study which emphasized the lack of development of lower-extremity thermal hypersensitivity with HWDS after sciatic nerve ligation [26].

#### 3.2.5. Lymphatic Function Effects

The contraction generated by rhythmical, ultra-low-frequency H-Wave therapy simulates voluntary contraction of smaller, slow twitch red fibers in skeletal muscle and smooth muscle fibers of lymphatic vessels [14,24]. Blum et al. further concluded that activation of smooth muscle fibers in lymphatic vessels facilitates movement of fluid and proteins from the extracellular space into the lymphatic system from areas of inflammation; rhythmic removal of lymphatic compression allows tissue recoil, creating a negative pressure within the interstitium, which corrects fluid shifts and ensures tissue homeostasis [24].

### 3.3. Clinical Studies

#### 3.3.1. Pain

Blum et al. (2006) analyzed the effects of HWDS on 1291 patients with neuropathic pain, assessing >25% pain relief as a positive outcome [23], which occurred in 62% with non-specific back pain, 65% in lower-extremity and 67% in upper-extremity cohorts. This observational study was subsequently extended by Blum et al. to include 6657 neuropathic pain patients, reporting overall positive outcomes in 78% [29]. Kumar et al. (1998) had previously studied patients with diabetic peripheral neuropathy, where 85% (12/14) who were treated with both amitriptyline and H-Wave therapy reported at least partial symptomatic relief compared to 56% treated with amitriptyline alone [30]; 36% of the combined treatment patients reported complete relief of symptoms. A similar cohort study by Julka et al. (1998) on the effects of HWDS for diabetic foot neuropathy patients demonstrated improvement in 76%, with an overall 34 ± 4% reduction in neuropathic pain [18]. Of the 24/34 cases that improved, 9 reported >50% symptomatic improvement. Earlier, Kumar et al. had also reported that for HWDS treatment of diabetic neuropathy, 83% (15/18) had improved neuropathic symptoms, with an average pain reduction of 52%, while 3 patients became completely asymptomatic [31]. A case report by Flatt had also reported that a 63-year-old disc rupture patient experienced complete restoration of pinprick, deep pressure, pain, and temperature sensation in his right lower extremity after six weeks of HWDS, following complete sensation loss after an accident 36 months prior to the start of treatment [32].

#### 3.3.2. Functionality

While HWDS has been associated with notable outcomes in diminishing pain, a meta-analysis by Blum et al. (2008) suggested that its largest effect size was through improvement in patient functionality [16]. A subsequent 2013 randomized controlled trial by Blum et al., applying HWDS during the post-operative phase of rotator cuff repair, reported significant improvements in shoulder range of motion after 45 and 90 days in both external and internal rotation; a number of H-Wave group patients advanced to physical therapy 2 weeks earlier than expected, something not observed in any sham-treated patient [27]. An observational study by Blum et al. tested the effects of HWDS on functionality in 1291 upper extremity, lower extremity, or back patients with neuropathic pain, reporting > 50% improvement in overall functionality based on a patient-reported questionnaire [H-Wave customer service questionnaire (HCSQ) including measurement of pain via 10-unit visual analog scale (VAS), reduction or discontinuation of pain medications, and improvement in function via ability to perform new activity] [23]; Blum subsequently reported that 79% improved in patient-reported functionality (HCSQ) after increasing the study population to 6530 patients [29].

#### 3.3.3. Pain Medication Use

A fairly robust 2006 study demonstrated significant reduction in pain medication usage by patients treated with HWDS for neuropathic pain in upper and lower extremity or back, assessed by patient-reported outcomes; >40% were able to reduce or completely eliminate pain medication use [23]. After expanding the study to 5329 patients with similar characteristics, 65% of patients were able to reduce or completely eliminate pain medication use, according to a patient-reported questionnaire [29].

## 4. Discussion

Given the inefficient efforts to treat pain over the past 4 decades, primarily with opioids, it has become increasingly critical to incorporate several differing modalities into a practical treatment plan, in order to address patient-specific aspects not covered by any single therapy [12,13]. Opioid pain medications hyperpolarize neurons at several levels along the spinothalamic pathway, culminating in decreased neuronal excitability and diminished pain sensation [5,33,34]. While chronic use significantly inhibits transmission of these stimuli to the brain, opioids are ineffective for eradicating the primary pain generators. In addition, opioids can also lead to an increase in pain sensitivity called hypoalgesia, due to alterations in dopamine tone [6].

Inflammation, impaired blood flow, and edema are prevalent nociceptive factors which diminish muscular function and exacerbate sensations of noxious stimuli and subsequent pain perception, thereby compromising patient well-being and decreasing functionality. Ignoring primary pain instigators has led to an unacceptable need for increased pain medication dosing, while inflammatory mediators continue to accumulate. It has become increasingly important to seek out and implement more effective treatment options which target the core etiologies for chronic musculoskeletal and neuropathic pain, in order to further decrease complications and treatment-related side effects. H-Wave^®^ therapy, where different frequencies of the waveform result in varying physiological effects, has the potential to hone in on separate aspects of the originators of pain.

Ultra-low-frequency HWDS appears to have a significant impact on blood vessels through rhythmic vasodilation, an effect manifested indirectly through skeletal muscle [14,15]. Induction of nitric oxide (NO) release by skeletal muscle stimulated by HWDS was found to be a principal vasodilatory component. Increased blood flow seems to be achieved by two separate mechanisms: (1) increasing diameter of blood vessels, and (2) increasing the number of vessels perfusing an area of healing (neovascularization). Chronic vasodilation has been shown to induce shear stress and wall tension upon blood vessels, creating an impetus for formation of new vessels [28,35]. An 11.5-fold HWDS-induced increase in blood vessels in rat lower-extremity muscles, in conjunction with the NO-mediated effects, synergistically enhances blood flow through the microcirculation via vasodilation and angiogenesis. Increased blood flow is certainly a principal component of ulcer healing, particularly in diabetics. With an overall diabetic foot ulcer healing rate of only 41%, the HWDS-induced average healing duration of 4 months to complete resolution warrants notice [25,36].

Lymphatic vessels similarly play a critical role in removal of inflammatory mediators from the interstitial space, where the accumulation of proteins may cause pain and decrease functionality. While nagging pain may often be the initial complaint, diminished function can subsequently be just as influential on patient reported outcomes, where chronicity of both has highly correlated to depression, anxiety, and subsequent cognitive decline [27,37]. Contraction of smooth muscle fibers in lymphatic vessels, generated by the rhythmical, ultra-low-frequency H-Wave therapy, activates and promotes movement of fluid and proteins from the extracellular space to improve the milieu of surrounding soft tissues [24]. This lymphatic mechanism, resulting in improved functionality, has been theorized to improve return-to-work time and enhance performance of activities of daily living (ADLs) [16]. Demonstration of increased range of motion with HWDS in rotator cuff reconstruction recovery, with quicker advancement to physical therapy, may be attributable to more rapid deep wound homeostasis resulting from enhanced blood flow and lymphatic vessel function [27].

The unique analgesic effect of high-frequency HWDS on nerve conduction adds a third dimension to recovery [6]. Nerves are responsible for transmitting stimuli to the brain, with afferent fibers eliciting action potentials by way of sodium channel pump activation. In chronic pain, such nerves become hypersensitized, leading to easier and earlier activation of these pathways the longer the tissue insult persists [38]. Commonly used forms of electrotherapy in the treatment of pain, like Transcutaneous Electrical Nerve Stimulation (TENS), offer some relief by way of gate theory, which is an overloading effect on ascending nerves to inhibit pain signals [24]. However, most current stimulator devices, including TENS, also activate large motor nerves, along with pain and sensory fibers, creating fatiguing, tetanizing contractions, and often only achieve short-term relief [17]. The non-tetanizing, non-fatiguing contractions generated by HWDS do not activate the same fibers and nerves as TENS, thereby alleviating many of the side effects associated with other types of electrical stimulators.

HWDS has been most readily studied as a treatment for neuropathic pain, where over one-third of diabetic patients suffer from the effects of peripheral neuropathy [18,31]. Three studies highlighted HWDS effects in diabetic peripheral neuropathy, resulting in at least partial relief in over 77% (51/66 patients) [18,30,31]. These findings were similar to a large observational study of 6591 patients with non-specific neuropathic pain, which reported >25% pain relief in 78% of the cohort [23]. Of note, both studies observed some pain relief in up to 37% of non-HWDS subjects, generated from the placebo effect of sham treatment. Interestingly, sub-group analysis demonstrated comparable positive results for carpal tunnel syndrome participants, providing suggestive effectiveness evidence supporting consideration of a larger range of diagnoses amenable to HWDS. Neuropathies naturally result in decreased function as well, where these studies noted that pain relief and improved functionality were achieved within a percentage point of each other.

Expansion of HWDS use, which is virtually without side effects, potentially translates to decreasing utilization of less effective and often harmful pain medications, where the convergence of addiction, tolerance, and dependence render the opioid drug class more harmful than beneficial in chronic pain management [39]. It might be helpful to screen patients for increased susceptibility to the harmful effects of opioids, more vigorously pursuing alternative treatments for those who may be more vulnerable for addiction. Blum et al. posed calculating a Genetic Addiction Risk Severity (GARS) on each chronic pain patient before beginning prolonged pain medication prescription [6]. In a recent study, the evaluation of pain clinic patients with GARS test and the Addiction Severity Index (ASI- Media Version V) scores revealed that scores equal to or greater than 4 and 7 alleles significantly predicted drug and alcohol severity, respectively [40]. Alternatively, HWDS has been shown to lower pain medication use in over 40–65% of neuropathic pain patients [23]. A meta-analysis of H-Wave therapy reported no adverse effects in more than 6000 patients [14].

This critical review included three randomized clinical trials, three observational studies, a case series, a case study, a meta-analysis, and six basic science papers. Despite being peer-reviewed and having some statistical significance, study weaknesses included heterogeneity and moderate risk of bias, while many results were based on patient reported outcomes, lacking other objective clinical findings. Future studies require better methodology, including more specifically defined patient populations, investigator independence, and sham/placebo devices which maximize blinding. Randomized controlled trials (preferably multi-center) remain the gold standard for best clinical evidence. Several higher-quality HWDS studies are currently under development which should eventually contribute to better understanding and expansion of specific indications for this promising pain treatment. Meanwhile, HWDS might be better understood and judiciously applied if carriers would carefully reassess the available evidence for effectiveness, particularly in light of the “opioid crisis”, since as a group most electrotherapies have been deemed to be “not scientifically sound” and therefore continue to generally not be covered. Exceptions should probably be considered for non-opioid multi-modal pain regimens for individual patients who show a positive response to initial (trial) treatment.

## 5. Conclusions

Chronic pain and decreased functionality are debilitating and complicated to treat, with past strategies proving to be ineffective and inefficient. H-Wave^®^ therapy has demonstrated some significant positive effects in a variety of diseases and disorders, resulting from several unique mechanisms which attack inflammation and pain generation at the source, with virtually no untoward side effects. HWDS has been shown in low- to moderate-quality studies to reduce reported pain, restore functionality, and lower pain medication use in a variety of musculoskeletal and neurological disorders; however, higher-quality research is needed to verify condition-specific applicability of HWDS, particularly as a stand-alone treatment. Pending larger cohort studies and multi-center trials, HWDS still has enough reasonable evidence to be considered on an individual patient basis for adjunctive use as a component of non-opioid multi-modal pain management, given its excellent safety profile and relative low cost.

## Figures and Tables

**Table 1:** Summary of characteristics of 16 selected HWDS studies.

Title	Author/Year	Study Type	HWDS Patient Numbers	Summary	Relevance	H-Wave^®^ Outcomes	Clinical Evidence Certainty
Coupling Genetic Addiction Risk Score (GARS) with Electrotherapy: Fighting Iatrogenic Opioid Dependence	Blum 2013 [6]	Policy Paper/ Pre-Clinical Study	_	Strategies to prevent opioid overdose deaths and attenuation of prescription abuse	_	Identifying patients with specific genes leading to opioid dependence in order to initiate non-pharmacologic H-Wave instead	_
Sciatic Ligation in Rats	Tsang, Tajkaishi 1998 [23]	Pre-Clinical Study	_	Electrotherapy effect on thermal hypersensitivity in rats after surgical ligation of sciatic nerve	Electrotherapy performed on 10 rats vs. control; withdrawal latency after heat application tested	Electrotherapy rats recovered latency to a greater extent	_
Innate Properties of H-Wave on Pain: A Hypothesis	Blum 2005 [24]	Pre-Clinical Study	_	Hypothesis behind H-Wave to limit inflammation by stimulating lymphatic smooth muscle	_	_	_
Innate Properties of H-Wave on Pain with Increased Functional Restoration: A Hypothesis	Blum 2005 [14]	Pre-clinical Study	_	Hypothesis behind H-Wave to limit inflammation by stimulating lymphatic smooth muscle	_	_	_
H-Wave device induces NO-dependent augmented microcirculation and angiogenesis	Blum 2008 [17]	Pre-Clinical Study	_	Hypothesis regarding H-Wave effects on NO production and angiogenesis	_	Deactivates sodium pumps within nerve fibers, leading to long-lasting analgesic effect	_
H-Wave induces arteriolar vasodilation in rat striated muscle	Smith 2009 [15]	Pre-Clinical Study	_	Measured H-Wave vasodilatory effects on rat arterioles in cremaster muscle	57 male rats, blocked with L-NAME, received HW at both 1 and 2 Hz	HW resulted increased blood flow between 26 and 62%	_
H-Wave Effects on Blood Flow and Angiogenesis in Longitudinal Studies in Rats	Smith 2011 [25]	Pre-Clinical Study	_	HW effect on hind limb blood flow	60 min daily of 2 Hz for 3 weeks	247% increase in blood flow above resting conditions; biopsy showed increased formation of new blood vessels in biceps femoris	_
Healing enhancement of chronic venous stasis ulcers utilizing H-Wave device therapy: a case series	Blum 2010 [26]	Case Series	3	Effect of HW on venous stasis ulcer healing	3 patients, different duration of ulcers, 30–60 min treatments at ultra-low; p1 twice daily; p2 once weekly; p3 once weekly until 9 months, then once daily	p1: healed after 3 months, p2: healed after 1 month, p3: healed after 9 months	Low
Resolution of a Double Crush Syndrome	Flatt 1994 [27]	Case Study	1	63 y/o patient slipped on ice, did the splits, and herniated L4-S1	2.25 min of ultra-low; 5 min of high 2x/wk for 6wks	36 months after reported injury, began treatment schedule; loss of pinprick, deep pressure, pain, and temperature sensation in RLE resolved after four weeks of HW and lumbar flexion/distraction	Low
Repetitive H-Wave device stimulation and program induces significant increases in the range of motion of post-operative rotator cuff reconstruction	Blum 2009 [28]	Double-Blind RCT	11	Range of motion and strength testing after HW therapy in post-op rotator cuff patients	22 patients, 11 sham; HW 1 h twice daily for 90 days post-op; evaluated on flexion, ER at side, ER at 90 degrees abduction, IR at side, IR at 90 degrees abduction	Significant improvement in both active ER and IR, no difference in strength testing; HW advanced more quickly through PT	Low
H-Wave meta-analysis	Blum 2008 [16]	Meta-Analysis	6535	Meta-analysis of H-Wave effect on pain, pain med use, increased functionality	HWDS twice daily for 90 days	Biggest improvement in functionality; moderate to strong effect in all	Moderate
H-Wave, a non-pharmacologic alternative to treatment of chronic soft tissue inflammation and neuropathic pain	Blum 2006 [29]	Observational Study	1291	Clinical study of H-Wave effect on pain, functionality, pain med usage in chronic inflammation	1291 patients used H-Wave on either LE, UE, or back for 2 to 6 weeks; previous moderate degree of pain 6–10/10	More than 60% reported over 25% pain reduction; functional improvement in over 50%; 40% reduced or completely eliminated pain meds	Moderate
H-Wave, a non-pharmacologic alternative to treatment of chronic soft tissue inflammation and neuropathic pain: Extended Study	Blum 2006 [30]	Observational Study	6774	Clinical study of H-Wave	6774 patients twice daily for 90 days	65% decreased need for pain med; 79% increased functionality; 78% symptomatic improvement	Moderate
Diabetic Peripheral Neuropathy: Amelioration of Pain with Transcutaneous Electrostimulation	Kumar, Marshall 1998 [31]	RCT	18	H-Wave electrotherapy on diabetic neuropathic pain vs. placebo	25–35 mA above 2 Hz as patient could tolerate	Symptomatic improvement in 15/18 patients; most pain relief was achieved by the third week; no side effects reported; one month f/u after stopping treatment yielded tendency for recurrence of symptoms	Moderate
Beneficial Effects of Electrical Stimulation on Neuropathic Symptoms in Diabetes Patients	Julka, Alvaro, Kumar 1998 [18]	RCT	54	H-Wave electrotherapy for diabetic neuropathic pain surveyed	Average twice daily for 35 min per treatment for 1.7 years	Over 34 +/− 4% reduction in pain; 2 point reduction on pain scale; 12/19 had reduction in LE swelling	Low
Diabetic Peripheral Neuropathy: Effectiveness of Electrotherapy and Amitriptyline for Symptomatic Relief	Kumar, Alvaro, Julka, Marshall 1998 [32]	RCT	14	Compared amitriptyline, amitriptyline + sham, amitriptyline + H-Wave on symptomatic relief of diabetic neuropathy	Amitriptyline 50 mg for all, 12 weeks of electrotherapy	12/14 patients had symptomatic improvement with H-Wave; 5 /14 with complete relief	Low

## Data Availability

Data is contained within the article.
